# Leisure education to leadership: Youth with physical disabilities’ experiences in South Africa

**DOI:** 10.4102/ajod.v12i0.1234

**Published:** 2023-11-30

**Authors:** Makhaya J. Malema, Marie E.M. Young, Lisa Wegner

**Affiliations:** 1 Department of Sports, Recreation and Exercise Science, Faculty of Community Health Sciences, University of the Western Cape, Bellville, South Africa; 2 Department of Occupational Therapy, Faculty of Community Health Sciences, University of the Western Cape, Bellville, South Africa

**Keywords:** leadership development, leadership skills, leisure, leisure education, youth with physical disabilities

## Abstract

**Introduction:**

Young people with physical disabilities frequently lack opportunities to develop their leadership potential. These include their ability to make decisions and be able to take charge of their leisure programmes. An argument is made that developing leadership skills for youth with physical disabilities can be facilitated by participating in leisure education programmes.

**Objectives:**

The objective of this study was to explore youth with physical disabilities’ perceptions of how leisure education can be used as a tool to develop their leadership.

**Method:**

This study used a descriptive qualitative research design to collect data using the purposive sampling method from 10 youths with physical disabilities aged 18 to 34 years in the Western Cape, South Africa. One-on-one interviews with semi-structured and open-ended questions were used to collect data for this study.

**Results:**

The findings of this study include 4 main themes and 11 sub-themes. This study’s findings showed that participants’ perceptions and experiences were evidence of leisure education being used to build their capacity as leaders within their society. Furthermore, their understanding of how they apply leadership opportunities is an encouraging moment for their development.

**Conclusion:**

Leisure education should be considered as a means to promote leadership in youth with physical disabilities in South Africa.

**Contribution:**

Knowledge and experience about youth with physical disabilities, their leisure education experiences and skills development during activities.

## Introduction

Leadership skills are a critical need for all youth, specifically in the context of youth with physical disabilities (YwPD) for this study. There are positive outcomes and influences that benefit a good leader among people in society (Kendellen et al. 2016). All leaders encounter challenges, specifically YwPD. Despite the known and unknown challenges, they have the ability to influence peers and form groups, which allows them to engage in leisure activities on their own terms. In this study, leadership development is applied in the context of YwPD’s ability to develop and influence relationships and skills among peers in a non-formal setting where leisure education programmes allow them to encourage each other (Shaikh, Bean & Forneris 2019). In an ideal world, YwPD would be independent of leisure service providers and take charge of their leisure programmes to address their needs and challenges. Youth with physical disabilities could act as peer models to each other with positive mutual goals when engaged in leisure programmes (Brymer & Gray 2006). This study forms part of a bigger project of using leisure education as an element for developing leadership capacity among YwPD within the South African context (Malema 2022).

Facilitating leadership-related qualities can be promoted using leisure education programmes such as camping (Martin 2018). The study was conducted on adolescents without disabilities in Southern Indiana, United States of America (USA). The camping programme duration was over 5 to 8 days for 7 weeks. Participants were allocated to an adventure camp (adrenaline activities) or a speciality camp (activities focusing on development). Daily activities for the adventure groups included ‘swimming, biking, creek hiking, canoeing, rock climbing, zip-lining, arts and crafts, archery, a mud pit, and a low rope course’ (p. 163). Speciality camp activities included ‘canoe camp, arts and crafts camp, wilderness camp, and leader in training (LIT) camp’ (Martin 2018:163). The results of the study indicated that social relationships, identity and self-image, agency and engagement, spirituality, ethicality and morality are significant areas of leadership development during the camp. Furthermore, Martin found that participants understood leadership according to the latest trends in leadership and positive youth development. Lastly, the participants of the study reported positive camp experiences and leadership development initiatives implemented (Martin 2018). The findings of the study contributed to knowledge transfer and behaviour change among participants through their experiences and perceptions of each camp programme.

Another example of leadership facilitation through leisure education programmes is Boettcher and Gansemer-Topf’s (2015) research on students without disabilities who participated in outdoor recreation programmes in Illinois, USA. Canoeing and kayaking trips as outdoor recreational activities were part of their skills development. The participants in the study reported communication and teamwork as part of their leadership development, as well as planning and organisation, adaptability and decision-making. The participants reported developing the role of empowerment, taking responsibility and individualised meaning. The findings of the study revealed that soft skills such as communication, competence and decision-making as elements of a leader can be nurtured and developed by engaging in leisure programmes (Boettcher & Gansemer-Topf’ 2015). Despite the literature mentioned above, no research has been conducted where leadership development for young people with disabilities was the intended outcome, specifically using leisure education as a tool. This study contributes to addressing the knowledge gap about leadership development for YwPD through leisure education.

### Developing leadership through leisure education

Leisure education is a tool that uses leisure activities, offers information and knowledge, and advances prospects for involvement in free-time activities among all people (Fukushina & Scwartz 2019; Sivan 2014). Beland (2008) argued that leisure service providers need to adapt their programmes, especially in communities, to meet the participation needs of people with disabilities. Malema, Young and Wegner (2022) reported that participating in leisure education programmes can allow leadership skills and capacity building among YwPD. Furthermore, by participating in leisure education programmes, people are able to explore how they are influenced and impacted on a personal level while they are part of the activities (Sivan 2008; Wilkinson, Kmiecik & Harvey 2020). Leisure education is argued to be an intermediate factor that could promote participants’ quality of life through engagement and sharing knowledge and information about activities that take place in communities (Datillo 2018; Sivan 2014). To obtain a feasible outcome from a leisure education programme, there would be a need to have specific, measurable outcomes guided by the appropriate frameworks to promote the programme’s objectives. This study adopts the leisure ability model and the self-management principle as guiding theoretical frameworks.

**FIGURE 1:**
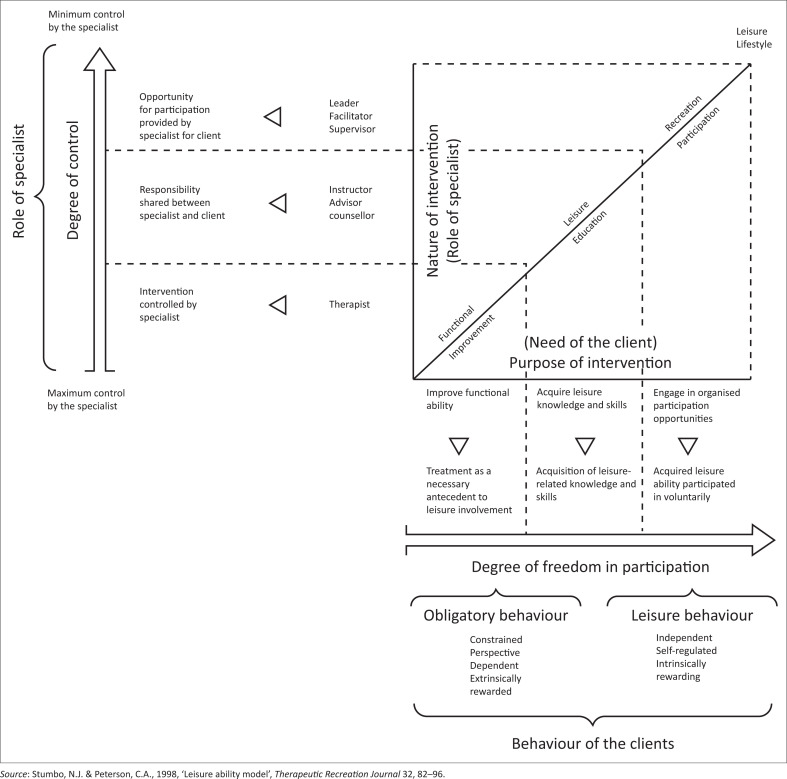
Leisure ability model.

Stumbo and Peterson (1998) developed a leisure ability model that focuses on therapeutic recreation contexts. The aim of the leisure ability model is to encourage an autonomous, active leisure lifestyle among people with disabilities. The leisure ability model comprises three key domains (see Figure 1): treatment, leisure education and recreation participation (Stumbo & Peterson 1998). The component of treatment is not the focus of this study; rather, leisure education and recreation participation domains are the focus of this study. The component of leisure education is central to this study because it is argued that it can develop leadership skills. In relation to the recreation participation component within the leisure ability model, YwPD will be able to learn skills that are appropriate to their development while they participate in the programmes. Figure 1 represents the process of the leisure ability model.

This study is based on the premise that leisure education and recreation participation can inspire YwPD to develop their leadership capacity. During recreation participation, YwPD demonstrate how their leisure engagement influences their choices and how this affects their behaviour. Leisure behaviour occurs during participants’ engagement in programmes. Through people’s leisure behaviour during activity repertoire, which includes attitude, values, needs that arise, knowledge, skills, experience and interest, YwPD can develop leadership skills. The outcome of developing leadership will lead to YwPD having a positive lifestyle. This is enhanced by their ability to engage with others, knowledge sharing and having a positive attitude when they are participating in leisure activities (Stumbo & Peterson 1998). In summary, the leisure ability model is the cornerstone for advancing an active, independent lifestyle for people with disabilities. People’s leisure choices and participation can facilitate the development of a positive and proper use of time (Hutchison & Robertson 2012). In this study, an argument is made that YwPD will move from the leisure education phase with knowledge and information and develop the necessary skills; they would then carry on participating freely in recreational activities and then develop leadership skills in the process.

Self-management as a framework for this study becomes relevant when considering YwPD as responsible for self-development and capacity building (Frantz & Rhoda 2021). The notion of self-management offers individuals the opportunity to capacitate themselves and have self-reliance and resilience based on the skills and resources that are at their disposal (Frantz & Rhoda 2021:3). Furthermore, people become more comfortable as they continue engaging in various activities, which results in increased self-efficacy (Clark & Schopp 2021:12). Cory, Dattilo and Williams (2006) stated that positive peer relationship has an influence towards youth’s personal perceptions of their own independence. Therefore, when considering leisure education and recreation participation from the leisure ability model and how these are managed by YwPD through their embracing of the self-management framework, developing leadership skills seems inevitable.

### Challenges from leisure and recreation programmes

Many young people lack suitable prospects to develop their leadership skills, which leads to them exercising decision-making and planning leisure activities on their own terms (Lazcano et al. 2021). Notably, leisure service providers do not offer their programmes with the intention of building their capacity; rather, they focus on pure participation so that they are not independent of the leisure and service providers (Lazcano et al. 2021). Dolva, Kleiven and Kollstad (2014) conducted a study using structured interviews on adolescents with Down syndrome in Norway. The findings of their study showed that adolescents did not have access to support and develop and participate actively in leisure programmes (Dolva et al. 2014).

In this study, the researchers posited that YwPD cannot be disregarded as leaders based on their disability. Young people’s creativity and courage should be protected so that they can facilitate social change (Wegner & Majee 2021). In this study context, creativity and social change are perceived as significant factors for a leader, to enable them to explore and influence their peers. A plea is made by Balt (2004) that all individuals have a role to play in the African continent’s development. Youth with physical disabilities’ contribution can be through embracing self-management skills. Self-management focuses on chronic health conditions and community integration (Frantz & Rhoda 2021), in which physical disability is considered within that context. According to Clark and Schopp (2021), the responsibility lies with individuals to ensure that they manage their chronic health proactively. It is therefore assumed that the critical skills of self-management can lead to leadership. Self-management is an important aspect of YwPD’s leadership development in this study.

This study aimed to gain an understanding of how to promote capacity building on YwPD using leisure education as a tool to develop leadership. The objective of this study was to explore YwPD’s perceptions of how leisure education can be used as a tool to develop their leadership. Therefore, this study attempts to respond to the research question: What are the perceptions held by YwPD about using leisure education as a tool to develop leadership?

## Methods

### Design

A qualitative descriptive research design was used to achieve the aim and objective of this study. The approach used in this study allowed the researchers to gather data and interpret the data based on the viewpoints of the participants (Shosha 2012).

### Participants

Ten participants who met the inclusion criteria were purposefully selected. Participants were recruited and selected in liaison with the Association for People with Disabilities (APD) in the City of Cape Town municipality in the Western Cape province, South Africa. These were that only YwPD aged 18 to 34 years were included in this study. Greeff (2011) proposed that purposeful sampling is useful to researchers to ensure relevant participants with the desired characteristics are identified for the study. Their physical disabilities were as a result of accidents, and some were born with a disability. Eight participants used wheelchairs for at least 5 years, whereas the two other participants used walking support aids.

### Inclusion and exclusion criteria

Only YwPD were recruited as participants in this study. Participants needed to be able to understand and speak English and comprehend information independently. Because of the lack of technical support, participants with additional disabilities (i.e. intellectual, hearing or visual) were excluded from this study. The support level required had the potential to misrepresent the participants’ perceptions, and important information could be lost in the process.

### Piloting

A pilot test of the interview questions was done, following an approach called ‘interviewing the investigator’ (Chenail 2014). This method requires that the researcher take up a role as a potential study participant and have a qualified qualitative researcher to conduct the interview as part of a mock-interview process (Chenail 2014). This allowed the researcher to amend and modify questions that were ambiguous.

### Data collection

South Africa adhered to the coronavirus disease 2019 (COVID-19) safety regulations, and the researcher ensured to follow the guidelines during data collection. The researcher made use of one-on-one interview method with semi-structured, open-ended questions with participants. Only one interview was conducted using Google Meet ™. Deakin and Wakefield (2013), in their research on the use of Skype interviewing, stated that online methods were a useful alternative for face-to-face interviews considering information and communication technology. Therefore, considering the climate over COVID-19, this method provided a unique and useful alternative while still ensuring thorough engagement between the researcher and participant. Ethical protocols were observed to ensure the participants’ rights and anonymity are preserved. Participants were interviewed until the data were deemed sufficient. ‘Data sufficiency in the study context refers to the point at which categories appear to guide the collected data without further modification’ (Varpio et al. 2016). Interview questions focused on the participants’ thoughts and views of leisure education and leadership development, as well as the importance that they assigned to leisure from their individual perspectives. A total of 10 individual interviews were conducted with the participants, and each interview lasted a minimum of 45 min. Interviews were conducted in a neutral venue that was comfortable and convenient for the participants. The researcher made notes and observations during interview sessions and probed participants throughout.

### Data organisation and analysis

Permission to record the interviews was sought from participants to allow a verbatim transcription. The first researcher checked the transcripts against the audio recording to ensure all contents were recorded and to promote the credibility of the data. An ATLAS.ti software was used during data coding and analysis. The data were presented following the thematic analysis approach. This method was appropriate to allow the researchers to identify, analyse and report themes within the data which will be useful when interpreting the findings (Braun & Clarke 2006). An open-ended coding technique was used wherein the researchers reflected on the process of immersing themselves in the data (Matthews & Kostelis 2011). To ensure there was no bias, the researchers consulted an independent co-coder to ensure proper themes and sub-themes were reported.

### Trustworthiness

To ensure the trustworthiness of the research, the guideline framework proposed by Schurink, Fouche, and De Vos (2011) was applied, which includes credibility, transferability and dependability. Credibility in this study was ensured by allowing engagement with participants during data collection, member checking and observations (Krefting 1991; Merriam 2009; Schurink et al. 2011). Member checking was used as the data collection and analysis were verified with the participants to ensure transparency and credibility. This allowed the researcher to probe participants for depth and understanding of the topic under investigation. Transferability in this study was achieved by thoroughly applying the research methodology and describing the literature context to ensure a detailed account is given (Krefting 1991; Merriam 2009; Schurink et al. 2011). Transferability refers to the extent to which the findings are applicable to comparable situations beyond the specific study environment, as determined by the degree of similarity. This study provided a dense and detailed description of the research setting and participants, thus enabling others to decide how the findings may apply in other similar settings. Dependability was ensured by using an independent co-coder to discuss the coding processes and reach a consensus to avoid bias and promote neutrality (Krefting 1991; Merriam 2009; Schurink et al. 2011).

### Ethical considerations

The study obtained ethical clearance and approval from the University of the Western Cape’s Biomedical Research Committee (BM20/2/1). Eligible participants were given informed consent forms to sign to confirm participation. All information was in English. Participants were able to speak, read and understand the language and comprehend information independently. Participation in this study was purely voluntary, and the researchers clarified any concerns and questions before data collection; this allowed participants to freely choose to be official participants. Pseudonyms were assigned to participants for anonymity purposes. One participant was advised to change the screen name for the online interview to avoid having their personal details captured in the recorded interview. Every research endeavour carries a certain level of risk. In this particular study, the researcher clarified that there was a potential for participants to experience discomfort and unease because of their daily challenges. A professional counselling and support session was put in place to ensure participants’ well-being is looked after. For this study, no participant required professional counselling. All participants were respected and valued for their experiences, and the researchers ensured that the environment where data were collected was comfortable, warm and welcoming.

## Results

The findings indicate how a leisure education programme can facilitate leadership skills development among YwPD. Table 1 reports the demographic information of the participants. Additionally, Table 2 illustrates the main findings where 4 themes and 11 sub-themes are reported. The main themes identified include leisure for YwPD, the nature of leisure education, leisure education learning areas and leadership opportunities.

**TABLE 1:** Demographic information of participants.

Participant	Gender	Age (years)	Highest qualification	Ethnic group
P1	Male	23	Diploma	African
P2	Male	31	Post Graduate degree	African
P3	Female	28	Certificate	Mixed-race
P4	Female	25	Degree	Afrikaans
P5	Female	33	Grade 12	African
P6	Female	24	Grade 9	Mixed-race
P7	Male	22	Busy studying a degree	Afrikaans
P8	Female	22	Certificate	Afrikaans
P9	Female	30	Grade 12	African
P10	Male	26	Degree	African

**TABLE 2:** Overview of the themes and sub-themes.

Theme	Sub-theme	Quotation
Leisure for YwPD	Importance of leisure for YwPD	‘I think it is important from my side so that people can see from me what I want, how I want to be treated. Because if I’m doing that thing, people will know how I want to be treated! People have to see from me to put into practice what I say … be treated that particular way.’ (P1)
	Role of leisure in development	‘I think it is important to formulate a programme that will benefit many people because now those people are learning about something in that programme and can end up with a valuable thing that they can use to reach their target life.’ (P2)
Nature of leisure education	Meaning of leisure education	‘During leisure education programmes, you learn through activities such as football, crickets, so on … you know, you’re learning in key sports. So, your learning, cooperation, you learn to get on through leisure.’ (P8)
	Purpose of leisure education programme	‘It calls us to come together and brings us together, especially people with disabilities, to sit together to find ways to attain our goals.’ (P6)
	Programme components	‘We can do programmes that will improve our limbs, e.g., stretch our arms, our legs. We can try to throw a ball at each other without hurting ourselves. Whatever activities introduced, youth with physical disabilities can be able to engage in them.’ (P4)
Leisure education learning areas	Active engagement	‘I think it is important for youth with a physical disability to engage in many activities to develop the skills.’ (YwPDP2)
	Motivation	‘I like to show other youth with disabilities that they can also do it. They must not feel ashamed of themselves and just come and see me doing it.’ (P6)
	Self-development	‘All of us have leadership abilities, some leadership skills, more so than most others. Suppose you like reading as a leisure activity, such activities enriching our lives. In that case, that’s one of the things I think people with physical disabilities can do to enhance their leadership qualities.’ (P7)
	Values and attitudes	‘The only disability out there is a bad attitude for sure. If you have a bad attitude, even if you’re physically disabled, normal, or differently-abled, a bad attitude will get you nowhere.’ (P7)
Leadership opportunities	Meaning of leadership development	‘For me to be a leader on my own, it’s the most important thing because I can take a leadership role and drive and motivate people to learn about everything.’ (P3)
	Leadership development platforms	‘I have learned something through leisure education, some people, especially people with disabilities, don’t have to stay in one place. They have to go out there and search for activities that will enhance their skills, and they will have some skills and abilities that will help them in life.’ (P2)

YwPD, youth with physical disabilities.

All the participants had been wheelchair users for at least 4 years (through car accidents, gunshots and some since birth). All participants resided in townships (semi-rural), which are classified as having low socio-economic disadvantage.

### Theme 1: Leisure for youth with a physical disability

Leisure is an essential phenomenon with many benefits. This theme describes the youth’s perspective on leisure, the importance of leisure for people with physical disabilities, the role of leisure, the value of belonging that results from being part of leisure activity programmes and what youth can offer during leisure programmes. Based on the youth’s perception, leisure is expressed as a human right available to all people, including those with physical disabilities.

#### Sub-theme: Importance of leisure for youth with physical disabilities

Participants expressed their perceptions that they are able to benefit from participating in leisure activities and gave an account of how they each experience leisure in a different manner. Participants reported the importance of leisure in this sub-theme, reporting that they recognised that leisure promotes healthy living and quality of life. One participant said:

‘It’s [*leisure participation*] really important for youth with physical disabilities because of anxiety, stuff, like there are all kinds of stuff … illnesses and so on, can affect you by lying around doing nothing.’ (P4, Female, 25)

Another participant expanded on this notion by stating:

‘If I take my situation, it’s basically what keeps me sane; if you’re going to stay indoors, that’s very bad for your mental health. And I think people with disabilities, I wouldn’t say, are more prone to mental health issues because I think everyone struggles with those kinds of things. Not just people with physical disabilities, but I think it is very important for anyone to partake in leisure activities, like, extremely important.’ (P9, Female, 30)

Participants were of the opinion that leisure has a significant role in their lives and that people should embrace the benefits of leisure activities. As reported by one participant in this sub-theme, mental health issues are common among people with disabilities. Therefore, one participant reported that to keep sane, leisure activities can be a mitigating factor for mental health issues. Therefore, it would be important to be actively involved in leisure activities to experience the positive benefits.

#### Sub-theme: Role of leisure in development

Leisure plays a significant role in the skills development of participants. The role of leisure has a direct impact on the development of participants. Youth with physical disabilities in this sub-theme reported:

‘I think it’s how they can learn that will make them be part of activities, not like sitting at home, not knowing what to do about the condition. Because now, this is not a basic thing. It’s not a normal life. It’s a disability. So now, we are not the same as other human beings. So now leisure activity will get us involved here so that these other people can know that I want to do this. I want to reach this. I want to go there for me to achieve this so that they can move forward in life.’ (P2, Male, 31)

‘… when there’s fun and joy in your leisure time … Helping others or doing activities and so on. It makes you want to do more so you won’t sit back with your coffee cup of coffee … you see an opportunity that I can go from here to there … this was fun, this is good … I enjoyed myself with this leisure time.’ (P4, Female, 25)

Leisure presents an opportunity for participants to develop and apply the skills acquired during leisure programmes. Participants illustrated this point as follows:

‘I think if it’s micro-skills or macro skills, or basic skills, or life skills, whatever the case might be, you are enriching people’s lives, you’re adding value to that person’s life …’ (P7, Male, 22)

‘You get good at activities just by doing what feels natural for you because practice makes perfect.’ (P8, Female, 22)

‘It’s not only your leisure programme stuff that learns, you as a participant also learn, but you also go different organisations, in the department of sports … all these places can promote people to increase your knowledge during leisure programmes.’ (P4, Female, 25)

The role of leisure in the lives of the participants in this study can facilitate and foster leadership skills development. As expressed by participants, this means that leisure can be experienced as a holistic approach to their overall development.

### Theme 2: Nature of leisure education

This theme describes how participants perceive the nature of leisure education. Participants described leisure education’s meaning, purpose and preferred programme components based on their experience and perception. The nature of leisure education is dependent on each participant’s perception and experience during activity programmes. Participants describe the manner in which the nature of leisure education is perceived in their context.

#### Sub-theme: Meaning of leisure education

In this sub-theme, participants describe their personal and subjective meaning and perceptions of leisure education, youth sated that Participants 2, 4 and 9 understand leisure education as a way of upskilling themselves and others. They expressed a shared view that during leisure education programmes, people get to enjoy the activities and simultaneously have an opportunity to learn something. They gave examples of reading time and cleaning the park, which could offer a learning opportunity as leisure time activities.

Participants’ expressions about leisure education indicate that it is not limited to a particular activity for them. It can be subjective according to individual perception. Participants further reported that anyone could be involved in leisure education activities for their benefit:

‘I must tell myself that it doesn’t mean that you can’t do anything just because you’re stuck in a wheelchair.’ (P6, Female, 24)

Furthermore, one youth with a physical disability stated:

‘You learn through activities like football, crickets, so on … you’re learning in these sports. You’re learning cooperation and much more.’ (P2, Male, 31)

Participants in this sub-theme expressed their perceptions of the meaning of leisure education based on their encounters and experiences. The participants expressed being engaged in a leisure programme that has meaningful results and a positive outcome.

#### Sub-theme: Purpose of leisure education programme

Leisure education can offer a continual learning process that offers participants diverse outcomes and benefits. Activities incorporated in leisure education programmes became meaningful to participants’ desired outcomes, as expressed by P1:

‘You can do whatever you want with your time with peace; just remember having fun and learning.’ (P1, Male, 23)

P7 added, to the notion above by listing meeting:

‘… new people, talk, interact, boost [*and*] live your life to the fullest [*when engaging in leisure activities*].’ (P7, Male, 22)

Another participant perceived the purpose of a leisure education programme as:

‘It calls us to come together and brings us together, especially people with disabilities, to sit together to find ways to attain our goals.’ (P6, Female, 24)

Leisure education programmes offer various learning and developmental opportunities in which participants can learn through their programme and activity engagement, as explained in the following two quotations:

‘It is very important because a person with skills could be sitting at home not knowing how much they can do or offer in the community. They don’t know how skilled they are, so maybe through a leisure education programme, all that can be revealed and nurtured …’ (P10, Male, 26)

‘My view is not the same as you, you want to reach your goal, and I want to reach my own goal like you want to become a sports person, and I want to become a teacher. So, when we are talking about our fields, you will learn something from my field, and I will learn something from your field …’ (P2, Male, 31)

Three participants emphasised the lifelong learning process in leisure education, stating that:

‘Your character becomes the fruit of your life, and people will look up to you and look to you for motivation and inspiration, and obviously, as a leader that leads them. So, I think it is very important to look at your character and attributes and how they develop during leisure programmes.’ (P9, Female, 30)

Additionally, P2 and P8 put an emphasis of a continued learning process which is facilitated by peer teaching. Furthermore, they reiterated the purpose of learning from each other so they can benefit their society.

When the opportunity arises for YwPD to build their capacity through leisure education programmes, they can use the skills acquired to impact their community, increasing inclusion.

#### Sub-theme: Programme components

Leisure programme components can vary depending on the intended outcome. Youth in this study wanted to be involved in active leisure education programmes that allowed them to move around and contributed to their overall physical health. Youth with physical disabilities expressed in this sub-theme that:

‘… programmes that will improve our limbs, e.g., stretch our arms, our legs. We can try to throw a ball at each other without hurting ourselves … for example, we give you maybe a meter or two, three meters, try to push yourself if you can, doing it independently.’ (P4, Female, 25)

Some participants suggested the following components as activities:

P5 suggested activities like swimming, soccer and cricket as leisure activities, and P8 mentioned darts, chess or computer games as ways to:

‘… just to have fun because in that you don’t even know that you’re learning, and you don’t even know that you’re … bettering yourself.’ (P8, Female, 22)

Some participants (P9 and P10) expressed advocating for leisure education programmes that can be undertaken in groups. The participants viewed this notion as a way to unite people and develop the capacity of a person and a team.

Participants of this study preferred activities that would promote independence, allowing them to take charge and build capacity, learn skills and implement them for themselves. Additionally, as reported in this theme, the programme component must cater to a diverse group and outcomes.

### Theme 3: Leisure education learning areas

Experiences gained from leisure education programmes varied according to the setting in which activities took place. Through leisure education programmes, different skills can be developed according to the activities participants are exposed to. Youth reported how active engagement, motivation, self-development, attitudes and values act as learning opportunities during leisure education programmes.

#### Sub-theme: Active engagement

Being part of a leisure education programme requires that participants be active in the process. This would make their participation in activities to be enjoyable and their development to become meaningful and impactful. Participants expressed this point as follows:

‘When people are getting engaged in many activities out there, I think that they learn, they learned certain things in life, that way they develop a variety of skills, it becomes part of a developmental phase.’ (P2, Male, 31)

Additionally,

‘So, being involved and part of a particular programme promotes active engagement meaning you are now engaged.’ (P4, Female, 25)

Expanding on this notion, one participant said:

‘Knowledge doesn’t come by itself. You know leisure or skill development whenever it feels it can uplift.’ (P5, Female, 33)

A practical example is given by one youth, who stated that:

‘… being a team member, seeing how the team captain vocalises and expresses their thoughts. So, you learn through that just being there. And you’re, I think, just being around the team, you see the team, how the team interacts and acts with each other …’ (P8, Female, 22)

In this sub-theme, participants reflected on the benefits of being part of the process of leisure education programmes. Participants shared their lived experiences that are important to understand how their involvement in leisure programmes can contribute to their skills development.

#### Sub-theme: Motivation

Being inspired as part of leisure education programmes was vital to participants. Motivation could stem from internal and external factors. Participants are reported saying:

‘Seeing someone who is doing that thing is motivation by itself, someone younger, doing something he can, whatever it is that is inspiration … with a right particularly the right mindset.’ (P1, Male, 23)

Additionally, one participant said:

‘Motivation is something that you believe in, what you are doing, what you want to … I think motivation is suitable for people with disabilities because it lifts their spirit. It makes them optimistic about what they want in life.’ (P3, Female, 28)

One participant expands on this, stating:

‘I like to show other youth with disabilities that they can also do it. They must not feel ashamed of themselves and just come and see me doing it.’ (P6, Female, 24)

Participants experience and perceive motivation differently, as reported by one youth, who stated that:

‘The concept of competition is great because it plays into your internal psyche … So, you try to better your opponent, you try to better yourself, you try to do better than before.’ (P8, Female, 22)

Another participant stated:

‘One can find courage by being more confident in who they are … I think that changes in your perspective and life. And that can be motivation, which is almost contagious. So, then you also want to inspire other people and then get them going.’ (P3, Female, 28)

One way of motivating people is through the sharing of information. A participant articulates this as:

‘When you have the information, you should share it with other people with disabilities, and this is the greatest motivation because people get inspired by the information shared with them. For example, we have a group where everyone informs everyone about the different activities that are taking place.’ (P10, Male, 26)

Participants in this sub-theme reported from first-hand experience how motivation is encountered during leisure education programmes from both internal and external factors.

#### Sub-theme: Self-development

Through leisure education, participants develop character. This sub-theme reports participants’ perceptions of the initiatives offered to them for their development. Participants stated that:

‘Self-development it’s something that you do by yourself to better yourself. So now, I think when you’re telling yourself that you want to develop certain skills in life, and you want to become a knowledgeable leader, and you can inspire someone out there in life.’ (P2, Male, 31)

Giving advice, one participant said:

‘If you fail, people must learn not to fall forever … Because there’s always another way to do better in life …’ (P3, Female, 28)

According to one youth participant:

‘Leadership in itself is self-development. We always have to sustain and maintain our skills … maintaining and staying motivated, especially in sports teams.’ (P5, Female, 33)

One participant suggests that:

‘Like if you read, the more you broaden your horizons, I think you’ll be open to new stuff to learn more things to learn new things.’ (P7, Male, 22)

Some participants with an expressive practical application of becoming self-developed stated that:

‘You need to interact with people to learn from them.’ (P8, Female, 22)

One youth participant added to this perspective:

‘It’s very important to upskill yourself and develop yourself as a leader.’ (P9, Female, 30)

In this sub-theme, participants demonstrated awareness about what it takes to develop themselves. It is important for youth in this study to realise where they are in their development to assess the level of intervention when necessary.

#### Sub-theme: Attitudes and values

The attitudes and values of both the participants and members of society influence their leisure engagement positively or negatively, as reported by participants in the following quotations:

‘… education is the key, through which leisure education can instil positive attitudes … you get to experience the basics about the influence of an attitude. Because if you can adapt your attitude, you can push yourself back if you’re not careful.’ (P1, Male, 23)

One youth is mindful of the impact of a leader’s ethics, morale and attitude, stating that:

‘What we are focusing on as the main goal is to bring their mindsets or attitudes or beliefs equal as yours just to lead each other around so that we can be equal into the thing together and focus on the same thing.’ (P4, Female, 25)

The phrase ‘it takes two to tango’ is amplified by one participant who realised that you get what you give, stating that:

‘I don’t call them a boy for all these things. I don’t use the word boy. I never use demeaning words no matter how young I think he is or how much younger they are to me, and I call them bras^1^.’ (P5, Female, 33)

One participant stressed:

‘The only disability out there is a bad attitude. If you have a bad attitude, even if you’re physically disabled, whether you are “normal,” or differently-abled, a bad attitude will get you nowhere …’ (P6, Female, 24)

Youth recognised the importance of the choice of words when addressing each other when engaging together in leisure programmes. Participants expressed that their values and attitudes during a leisure programme and in their social interaction influence their participation and the people around them.

### Theme 4: Leadership opportunities

Given the opportunity, YwPD can become leaders within their societies and peer circles. Through leisure education programmes, leadership roles are assumed and undertaken when assigned. Leadership positions might vary in different circumstances. The sub-themes reported in this theme give context based on YwPD’s perceptions.

#### Sub-theme: Meaning of leadership development

The meaning of leadership could vary according to each leader’s role and the type of leadership each person aligns themselves with. In this sub-theme, YwPD reported what leadership development means for them as follows:

‘I think leadership development, it’s whereby you lead, and you lead people. It’s whereby you lead people so they can develop certain skills in life to reach their potential.’ (P2, Male, 31).

‘It’s about transferring your skills and knowledge to the other person,’ (P3, Female, 28)

On the same thinking, one participant said that:

‘You can be a leader by helping others, showing others how to do stuff without having money or something like that. You can start something up from scratch.’ (P4, Female, 25)

Reporting further on this sub-theme, one youth said Participants 5, 7 and 9 reiterated that leadership involves leading a group of people, with a common goal. The participants highlighted the need to listen to the needs of the followers, teaching them necessary skills and serving the greater purpose and goal. More importantly, P9 mentioned a servant type of leadership:

‘It’s taking the step of leading the people, listening to their needs, understanding their needs, and how you can help them get these needs sorted … out’ (P5, Female, 33)

‘… leadership development is, I think, is to teach people about their skills, the micro-skills, or the macro skills, to develop leadership qualities.’ (P7, Male, 22)

‘… leadership to me is more about serving, and then obviously developing that person’s character, leading a group of people, but also primarily serve them.’ (P9, Female, 30)

Leadership has personal meaning for everyone, as stated by participants in this sub-theme. Youth experience leadership as leading a group, serving, transferring skills and helping them build their capacity.

#### Sub-theme: Leadership development platforms

The notion that youth would be able to build themselves up if offered appropriate leisure opportunities was expressed by participants under this sub-theme. This study suggests that offering leisure opportunities to youth could enhance their leadership potential. Youth with physical disabilities report on their leadership development platforms, saying:

‘… starts in the community, open a group programme, be the leader in your community, open something, and be a leader.’ (P1, Male, 23)

‘People with disabilities don’t have to stay in one place. They have to go out there and search for activities that will enhance their skills, and they will have some skills and abilities that will help them in life, which can be through leisure education programmes.’ (P2, Male, 31)

Adding to that, one youth said:

‘These guys need a good role model to change their way of thinking … we have to stand up as the township’s leadership and try to bring new ideas.’ (P6, Female, 24)

Some participants expressed that another platform for developing leadership can be reinforcement strategies:

‘I don’t think people understand the power of positive word through reinforcement, its magic …’ (P5, Female, 33)

Additionally:

‘It’s important to get out of your comfort zone. The world owes you nothing … you can, build people skills, meet new people socialise, build confidence, build self-esteem, build your ego and your development …’ (P7, Male, 22)

One youth participant raised an important point:

‘There should be a mixture of people, but we don’t want to be excluded. A disabled person is normal just like any other person with rights, that’s how we develop, through integrated programmes.’ (P10, Male, 26)

Opportunities for leadership development can be implemented through communities. As reported by youth, development platforms can be created by meeting new people, integrating programmes and stepping out of one’s comfort zone.

## Discussion

This study reported here aimed to explore YwPD’s perspectives on how leisure education can be used as a tool to develop leadership. Findings showed that participants recognised the importance of leisure, understood leisure education programmes and learning areas within leisure education programmes and acknowledged the available leadership opportunities afforded through leisure education participation. Their participation in leisure education programmes is driven by their desire to demonstrate their ability to be part of activities within their communities, demonstrating their skills and how they develop through leisure activities. Such a positive attitude displayed by youth in this study resonates with self-management as a platform for growth and development (Frantz & Rhoda 2021). The concept of self-management is based on the premise that people can proactively become independent to manage their day-to-day chronic conditions. This includes three essential tasks: medical management, behavioural management and emotional management. The essential tasks are complemented by the five core skills: problem-solving, decision-making, resource utilisation, partnership with providers, and acting (Frantz & Rhoda 2021). The domains and skills within self-management (e.g. decision-making; planning; social persuasion; locating, accessing and utilising resources; and assisting people in developing relationships) link with leadership skills in this study.

This study found that leisure education can offer capacity development in YwPD through various activities. The findings suggest that leisure education can enable YwPD to become active role players during their leisure time. Devine (2004) acknowledged that leisure can challenge a person’s existing character and identity. Stumbo and Peterson (1998) emphasised that getting involved in leisure education programmes instils valuable skills that can positively influence your future participation in these programmes. Datilto (2008) suggested that allowing youth to transfer skills and lessons learned outside of their leisure education programmes can be helpful to the participants by closing the gap between knowing how to participate and where to participate, ultimately leading to independence and self-determination in leisure behaviour and choices.

The context within which leisure education is created and executed is vital to attaining leisure-related skills, knowledge, attitudes and perceptions (Yankholmes & Lin 2012). Study participants experienced active engagement, motivation, self-development, and attitudes and values that enabled their learning and developmental process to become leaders. Participants showed interest in being actively engaged in leisure programmes offered in their communities, including being part of the planning process (Sivan 2014, 2017; Sivan & Chan 2012). Participants were motivated during leisure programmes when they saw other people taking part in the activities, which contributed to participants developing their leisure preferences and ensuring fun and enjoyment during leisure programmes (Sivan 2014, 2017, Sivan & Chan 2012). Participants experienced self-development through their willingness, abilities and interactions with others to develop their leadership capacities (Frantz & Rhoda 2021; Sivan 2014, 2017; Sivan & Chan 2012). Participants in this study valued their peers, and their attitudes did not change based on the age or culture of their peers, which aim to encourage people to appreciate the importance of leisure, discover its meaning and think positively about it (Sivan 2014, 2017, Sivan & Chan 2012).

Lazcano et al. (2021) stated that young people in general do not have sufficient opportunities to develop their capacity to make decisions and organise their activities, regardless of the context of the programmes organised. Datillo (2018) suggested a generic framework that would make leisure activities available and user-friendly to all people for meaningful participation. It is understood that leisure education presents an opportunity to promote the leisure capacity of participants through training, which can facilitate and increase their leisure competence (Datillo & Murphy 1991; Sivan & Stebbins 2016). Brymer and Gray (2006) stated that a leader requires soft and conceptual skills such as social matters, psychology, communication, sound judgement and creativity. Being developed holistically as a leader enables YwPD in this study to thrive in any challenge during leisure time.

The researchers argue that YwPD can embrace and develop the fundamentals reported in Fulthorp and D’Eloia’s (2015) study, which investigated critical competencies for human resource municipal recreation agencies in California, USA. Competencies such as: (1) the ability to make ethical decisions, (2) the ability to act professionally, (3) the ability to work well with people, (4) the ability to communicate clearly with others, (5) enthusiasm and a positive attitude and (6) the ability to work in a team environment are important characteristics of an effective leader (Fulthorp & D’Eloia 2015). As Jordan and Ramsing’s (2017) reiterated, leaders’ attitudes, words and actions profoundly affect their followers. Youth in this study recognised the importance of acquiring leadership skills during leisure programmes as beneficial to their lives. This suggests that youth can benefit from equal opportunities to fulfil their roles as leaders for their peers.

It is understood that youth encounter challenges while experiencing and developing themselves as leaders. The study’s findings align with Hutchison and Robertson’s (2012) proposition that leisure education programmes can prepare YwPD’s transitions in society as they develop leadership skills. This transition is important because it will enable YwPD to navigate their daily lives and livelihoods and deal with challenges as leaders. Study participants reported experiences of marginalisation because of a lack of resources, which hindered their ability to develop leadership skills. The skills developed from leisure education create opportunities for choosing from various activities (Cory et al. 2006; Datillo 1999; Datillo, Williams & Cory 2003; Marsden 2010). Wilkinson et al. (2020) noted that young people’s socialisation into and through leisure is significantly influenced by external forces such as family, friends and the development of global trends like technology. Wegner and Majee (2021) proposed that young people with the capacity and skills to manage themselves are better equipped to confront the challenges they face in changing environments. This study contends that YwPD are capable of becoming leaders, and their perceptions are an indication that they build their capacities and develop leadership skills.

## Conclusion

### Study limitations

There is a lack of resources and knowledge in South Africa regarding leisure education, which, in the context of this study, is perceived as a limitation of this study. Availability of resources and knowledge consists of being more specific about the kind of resources and knowledge required. Despite limited resources and knowledge about leisure education programmes, the results of this study lay the foundation for capacity development and youth with disabilities’ potential within the South African context.

### Recommendations and practical implications

Future research is encouraged to consider adapting leisure education with the intention of developing skills for the YwPD. It is further recommended that leisure education service providers, for example, the Department of Sport, Culture and Recreation (national, provincial and local level) and non-governmental organisations (NGOs) like *Let’s Play*, collaborate with youth with disabilities during leisure to ensure that programme planning becomes relevant to participants. The implication of this study is that the leisure service providers are encouraged to provide programmes that allow participants to develop skills while engaged in the activities. For leisure education programmes to be more meaningful, youth should be at the forefront of programme planning to ensure their needs are represented. And considerations should include the nature of the disability that youth live with to facilitate peer relationships and role modelling.

## Footnotes

1 Bra(s) is a slang word in South Africa, used as a sign of respect to a male gender perceived to be worthy of the respect.

## References

[CIT0001] Balt, M., 2004, ‘Youth leadership development programs in Africa: Assessing two case studies’, Doctoral dissertation, Stellenbosch University, Stellenbosch, viewed 05 November 2020, from http://hdl.handle.net/10019.1/50233.

[CIT0002] Beland, R., 2008, ‘The use of leisure time’, in T. Oakland & P.L. Harrison (eds.), *In adaptive behaviour assessment system-II*, pp. 159–178, Academic press, San Diego, CA.

[CIT0003] Boettcher, M.L. & Gansemer-Topf, A.M., 2015, ‘Examining leadership development through student leader outdoor recreation training’, *Recreational Sports Journal* 39(1), 49–58. 10.1123/rsj.2014-0034

[CIT0004] Braun, V. & Clarke, V., 2006, ‘Using thematic analysis in psychology’, *Qualitative Research in Psychology* 3(2), 77–101. 10.1191/1478088706qp063oa

[CIT0005] Brymer, E. & Gray, T., 2006, ‘Effective leadership: Transformational or transactional?’, *Journal of Outdoor and Environmental Education* 10(2), 13–19. 10.1007/bf03400835

[CIT0006] Chenail, R., 2014, ‘Interviewing the investigator: Strategies for addressing instrumentation and researcher bias concerns in qualitative research’, *The Qualitative Report* 16(1) 255–262. 10.46743/2160-3715/2011.1051

[CIT0007] Clark, B. & Schopp, L., 2021, ‘The case for self-management’, in J. Frantz, L. Schopp & A. Rhoda (eds.), *Self-management in chronic illness: Principles, practice and empowerment strategies for better health*, pp. 11–32, Springer, Geneva.

[CIT0008] Cory, L., Dattilo, J. & Williams, R., 2006, ‘Effects of a leisure education program on social knowledge and skills of youth with cognitive disabilities’, *Therapeutic Recreation Journal* 40(3), 144–164.

[CIT0009] Datillo, J., 1999, *Leisure education program planning: A systematic approach*, No. Ed. 2, Venture Publishing Inc., State College, PA.

[CIT0010] Dattilo, J., 2008, *Leisure education program planning: A systematic approach*, Venture, State College, PA.

[CIT0011] Dattilo, J., 2016, ‘A balanced and systematic leisure education service delivery model: Connections to Eastern and Western perspectives’, *World Leisure Journal* 58(3), 179–192. 10.1080/16078055.2016.1162842

[CIT0012] Dattilo, J., 2018, ‘An education model to promote inclusive leisure services’, *The Journal of Park and Recreation Administration* 36(2), 177–195. 10.18666/jpra-2018-v36-i2-8447

[CIT0013] Dattilo, J. & Murphy, W.D., 1991, *Leisure education program planning: A systematic approach*, Venture Publishing Inc., State College, PA.

[CIT0014] Dattilo, J., Williams, R. & Cory, L., 2003, ‘Effects of computerised leisure education on knowledge of social skills of youth with intellectual disabilities’, *Therapeutic Recreation Journal* 37(2), 142–155.

[CIT0015] Deakin, H. & Wakefield, K., 2014, ‘Skype interviewing: Reflections of two PhD researchers’, *Qualitative Research* 14(5), 603–616. 10.1177/1468794113488126

[CIT0016] Devine, M.A., 2004, ‘Being a “Doer” instead of a “Viewer”’: The role of inclusive leisure contexts in determining social acceptance for people with disabilities’, *Journal of Leisure Research* 36(2), 137–159. 10.1080/00222216.2004.11950017

[CIT0017] Dolva, A.S., Kleiven, J. & Kollstad, M., 2014, ‘Actual leisure participation of Norwegian adolescents with Down syndrome’, *Journal of Intellectual Disabilities* 18(2), 159–175. 10.1177/1744629514523158 24515503

[CIT0018] Frantz, J. & Rhoda, A., 2021, ‘Overview of self-management’, in J. Frantz, L. Schopp & A. Rhoda (eds.), *Self-management in chronic illness: principles, practice and empowerment strategies for better health*, pp. 3–9, Springer, Geneva.

[CIT0019] Fukushima, R.L.M. & Schwartz, G.M., 2021, ‘Design, evaluation and outcomes of leisure education programs’, *LICERE-Journal of the Interdisciplinary Graduate Program in Leisure Studies* 24(1), 106–129. 10.35699/2447-6218.2021.29498

[CIT0020] Fulthorp, K. & Eloia, M.H., 2015, ‘Managers’ perceptions of entry-level job competencies when making hiring decisions for municipal recreation agencies’, *Journal of Park and Recreation Administration* 33(1), 57–71.

[CIT0021] Greeff, M., 2011, *Information collection: Interviewing Research at grassroots: For social sciences and human service professions*, vol. 4, pp. 341–375, viewed 29 September 2023, from https://about.google/brand-resource-center/trademark-list/.

[CIT0022] Hutchison, B. & Robertson, B., 2012, ‘Leisure education: A new goal for an old idea whose time has come’, *American Society of Social Pedagogy* 19, 127–139.

[CIT0023] Jordan, D.J. & Ramsing, R., 2017, *Leadership in leisure services: Making a difference*, Venture Publishing, Urbana, IL.

[CIT0024] Kelland, J., 1996, *Community therapeutic recreation service*, Alberta Hospital Edmonton, Alberta.

[CIT0025] Kendellen, K., Camire, M., Bean, C.N. & Forneris, T., 2016, ‘Facilitators and barriers to leadership development at a Canadian Residential Summer Camp’, *Journal of Park and Recreation Administration* 34(4), 36–50. 10.18666/jpra-2016-v34-i4-6514

[CIT0026] Krefting, L., 1991, ‘Rigor in qualitative research: The assessment of trustworthiness’, *The American Journal of Occupational Therapy* 45(3), 214–222. 2031523 10.5014/ajot.45.3.214

[CIT0027] Lazcano, I., Madariaga, A., Romero, S. & Kleiber, D., 2021, ‘The importance of self-management in the leisure activities of young people’, *World Leisure Journal* 64(1), 23–34. 10.1080/16078055.2021.1937303

[CIT0028] Malema, M.J., 2022, ‘Guidelines for leadership development using leisure education as a tool for youth with physical disabilities in South Africa’, Doctoral thesis, University of the Western Cape, South Africa. 10.4102/ajod.v11i0.1073 PMC9724115 36483845

[CIT0029] Malema, M.J., Young, M.E.M. & Wegner, L., 2022, ‘Leisure programmes that promote leadership amongst youth with, and without disabilities: A scoping review’, *African Journal for Physical Activity and Health Sciences (AJPHES)* 28(1), 47–62. 10.37597/ajphes.2022.28.1.4

[CIT0030] Marsden, S., 2010, *Effectiveness of participation in a leisure education program on knowledge of aspects of community reintegration for individuals who have recently sustained spinal cord injuries*, viewed 05 November 2020, from https://tigerprints.clemson.edu/all_theses/814.

[CIT0031] Martin, K., 2018, ‘Summer camp youth leadership development: An investigation of adolescents’ perceptions of best practices’, *Journal of Youth Development* 13(1–2), 161–182. 10.5195/jyd.2018.536

[CIT0032] Matthews, T.D. & Kostelis, K.T., 2011, *Designing and conducting research in health and human performance*, John Wiley & Sons, New York, NY.

[CIT0033] Merriam, S.B., 2009, *Qualitative research: A guide to design and implementation*, Jossey Bass, Hoboken, NJ.

[CIT0034] Schurink, W., Fouche, C.B. & De Vos, A.S., 2011, ‘Qualitative data analysis and interpretation’, in A.S. De Vos, H. Strydom, C.B. Fouche & C.S.L. Delport (eds.), *Research at grassroots: For the social and human services professions*, 4th edn., pp. 397–423, Van Schaik Publishers, Pretoria.

[CIT0035] Shaikh, M., Bean, C. & Forneris, T., 2019, ‘Youth leadership development in the Start2Finish running & reading club’, *Journal of Youth Development* 14(1), 112–130. 10.5195/jyd.2019.674

[CIT0036] Shosha, G.A., 2012, ‘Employment of Colaizzi’s strategy in descriptive phenomenology: A reflection of a researcher’, *European Scientific Journal* 8(27), 31–43.

[CIT0037] Sivan, A., 2008, ‘Leisure education in educational settings: From instruction to inspiration’, *Society and Leisure* 31(1), 49–68. 10.1080/07053436.2008.10707769

[CIT0038] Sivan, A., 2017, ‘Leisure education in schools: Challenges, choices and consequences’, *World Leisure Journal* 59(1), 15–21. 10.1080/16078055.2017.1393871

[CIT0039] Sivan, A. & Chan, D.W., 2012, ‘Leisure education in schools from students’ perspectives: The case of Hong Kong’, *World Leisure Journal* 54(1), 26–37. 10.1080/04419057.2012.668039

[CIT0040] Sivan, A. & Stebbins, R.A., 2016, ‘Leisure education: Definition, aims, advocacy, and practices–are we talking about the same thing (s)?’, in A. Sivan & R.A. Stebbins (eds.), *In leisure education: A cross-national view*, pp. 3–17, Routledge, New York.

[CIT0041] Stumbo, N.J. & Peterson, C.A., 1998, ‘Leisure ability model’, *Therapeutic Recreation Journal* 32, 82–96.

[CIT0042] Varpio, L., Ajjawi, R., Monrouxe, L.V., O’Brien, B.C. & Rees, C.E., 2016, ‘Shedding the cobra effect: Problematizing thematic emergence, triangulation, saturation and member checking’, *Medical Education* 51(1), 40–50. 10.1111/medu.13124 27981658

[CIT0043] Wegner, L. & Majee, W., 2021, ‘Self-management in youth’, in J. Frantz, L. Schopp & A. Rhoda (eds.), *Self-management in chronic illness: Principles, practice and empowerment strategies for better health*, pp. 99–111, Springer, Geneva.

[CIT0044] Wilkinson, S., Kmiecik, K. & Harvey, W., 2020, ‘Community connections: Leisure education through afterschool programming’, *Leisure* 44(3), 421–439. 10.1080/14927713.2020.1780935

[CIT0045] Yankholmes, A.K.B. & Lin, S., 2012, ‘Leisure and education in Ghana: An exploratory study of university students’ leisure lifestyles’, *World Leisure Journal* 54(1), 58–68. 10.1080/04419057.2012.668044

